# Effects of Obesity on Perivascular Adipose Tissue Vasorelaxant Function: Nitric Oxide, Inflammation and Elevated Systemic Blood Pressure

**DOI:** 10.1159/000443885

**Published:** 2016-02-25

**Authors:** Reza Aghamohammadzadeh, Richard D. Unwin, Adam S. Greenstein, Anthony M. Heagerty

**Affiliations:** ^a^Institutes of Cardiovascular Sciences, Faculty of Medical and Human Sciences, The University of Manchester, Manchester, UK; ^b^Human Development, Faculty of Medical and Human Sciences, The University of Manchester, Manchester, UK; ^c^Institutes of Centre for Advanced Discovery and Experimental Therapeutics, Central Manchester University Hospitals NHS Foundation Trust, Manchester Academic Health Sciences Centre, Manchester, UK

**Keywords:** Obesity, Perivascular adipose tissue, Contractility, Endothelium, Nitric oxide, Superoxide dismutase

## Abstract

**Introduction:**

Perivascular adipose tissue (PVAT) surrounds most vessels in the human body. Healthy PVAT has a vasorelaxant effect which is not observed in obesity. We assessed the contribution of nitric oxide (NO), inflammation and endothelium to obesity-induced PVAT damage.

**Methods:**

Rats were fed a high-fat diet or normal chow. PVAT function was assessed using wire myography. Skeletonised and PVAT-intact mesenteric vessels were prepared with and without endothelium. Vessels were incubated with L-NNA or superoxide dismutase (SOD) and catalase. Gluteal fat biopsies were performed on 10 obese and 10 control individuals, and adipose tissue was assessed using proteomic analysis.

**Results:**

In the animals, there were significant correlations between weight and blood pressure (BP; r = 0.5, p = 0.02), weight and PVAT function (r = 0.51, p = 0.02), and PVAT function and BP (r = 0.53, p = 0.01). PVAT-intact vessel segments from healthy animals constricted significantly less than segments from obese animals (p < 0.05). In a healthy state, there was preservation of the PVAT vasorelaxant function after endothelium removal (p < 0.05). In endothelium-denuded vessels, L-NNA attenuated the PVAT vasorelaxant function in control vessels (p < 0.0001). In obesity, incubation with SOD and catalase attenuated PVAT-intact vessel contractility in the presence and absence of endothelium (p < 0.001). In obese humans, SOD [Cu-Zn] (SOD1; fold change −2.4), peroxiredoxin-1 (fold change −2.15) and adiponectin (fold change −2.1) were present in lower abundances than in healthy controls.

**Conclusions:**

Incubation with SOD and catalase restores PVAT vasorelaxant function in animal obesity. In the rodent model, obesity-induced PVAT damage is independent of endothelium and is in part due to reduced NO bioavailability within PVAT. Loss of PVAT function correlates with rising BP in our animal obesity model. In keeping with our hypothesis of inflammation-induced damage to PVAT function in obesity, there are lower levels of SOD1, peroxiredoxin-1 and adiponectin in obese human PVAT.

## Introduction

Obesity has a profound effect on the cardiovascular risk profile. Specifically, obesity often co-exists with hypertension in the context of the metabolic syndrome [1], and the understanding of this relationship has been substantially advanced by studies into the vasoactive properties of the adipose tissue surrounding blood vessels, known as perivascular adipose tissue (PVAT). Soltis and Cassis [2] were the first to show that PVAT attenuates arterial vasoconstriction. More recently, the vasorelaxant effect of PVAT has been demonstrated in both resistance and conduit arteries and appears to utilise multiple physiological mechanisms. There is evidence for both endothelial-independent and endothelial-dependent pathways depending on the species, vessel bed and disease of interest. Thus far, hydrogen sulphide [3], angiotensin 1-7 [4,5], adiponectin [6] and methyl palmitate [7] have all been implicated in the vasorelaxant effect. We have shown that in subcutaneous human tissue from healthy participants, adiponectin release from PVAT acting via nitric oxide (NO) is the predominant mediator of the vasorelaxant effect on small arteries [6]. We also observed that in obese patients with metabolic syndrome there was complete loss of the vasoactive properties of PVAT [6]. More recently, we have shown that the PVAT vasorelaxant effect can be restored 6 months following surgery and have demonstrated a significant reduction in TNF-α and macrophages within PVAT following weight loss to support the theory of inflammatory damage to PVAT function in obesity [8]. In this follow-on study, we used a proteomic approach to examine, for the first time, molecular changes to subcutaneous PVAT in obese patients compared with healthy participants. We used the findings to advance the ‘inflammation-induced damage’ hypothesis to account for damage to PVAT function in obesity and validated this in an animal model of diet-induced obesity. Moreover, we have shown correlations between PVAT function and blood pressure (BP) in obese rats, which highlights the potential relevance of PVAT studies to clinical practice.

## Materials and Methods

### Animal Studies

#### Animal Model Development

Four-week-old male Sprague Dawley rats were purchased from Charles River (Oxford, UK). After a 1-week acclimatisation period, the rats were divided into 2 groups (3 per cage, changed to 2 per cage after 2 months when obese rats could not be physically housed in the same cages due to their increase in size), receiving either a high-fat diet (Special Diets Services, Western RD; 4.24 kcal/g AFE; 35% of energy derived from fat; n = 16) or standard laboratory chow (3.29 kcal/g AFE; n = 6) for 3 months in a standard experimental animal laboratory, illuminated from 6:30 a.m. to 6:30 p.m. at a temperature of 22 ± 1°C. The protocol was approved by the Animal Experimentation Committee of the Medical Faculty of The University of Manchester (UK). The animals had free access to food and water during the experiment.

Body weight and BP were monitored once a week, and the consumption of feed was monitored daily using a standard laboratory table scale. At the time of sacrifice, the animals were weighed, their BP was recorded (LE5002 Storage Pressure Meter, Panlab Harvard Apparatus) and blood glucose was analysed using a portable glucose monitoring system (Ascensia® CONTOUR® blood glucose monitoring system, Bayer HealthCare).

#### Vessel Preparation and Myography

The mesenteric bed was immediately removed after animal sacrifice, and isolated mesenteric vessels with an average internal diameter of 250 μm were dissected and prepared as segments with intact PVAT and segments without PVAT. The vessels were studied using a multi-wire myograph system (Model 610 M, version 2.2; Danish Myo Technology) and analysed with ADInstruments Chart™ 5 (version 5.5.1). The vessels were bathed in physiologic saline solution (PSS; as above), maintained at 37°C and gassed with a mixture of 95% air and 5% CO_2_. The vessels were normalised and their viability was checked by the addition of PSS containing 60 mM of KCl, following which concentration-response curves to norepinephrine (NE; A0937; Sigma-Aldrich) were constructed. To assess the effect of free radical scavengers on the obese PVAT, vessels from the obese cohort were incubated with superoxide dismutase (SOD; 100 U/ml; Sigma-Aldrich) and catalase (100 U/ml, Sigma-Aldrich) for 45 min, following which another NE concentration-response curve was constructed. Similarly, vessels were incubated with L-NNA (10^-4^M, 45 min; Sigma-Aldrich) to assess NO bioavailability within PVAT. When required, removal of the endothelium was performed by a careful brushing technique using a 40-μm-diameter wire (the same as that used for myography) under visualisation by microscopy.

### Human Studies

#### Study Population

Ten patients with obesity and 10 control participants gave full written informed consent prior to their participation in the study, which was approved by the Local Research Ethics Committee. Fasting venous blood samples were taken to assess glycaemic and lipid profiles and inflammatory markers. After 15 min of rest, BP was measured in a sitting position using a semiautomatic machine (OMRON 705CP; White Medical) with the mean of 3 readings recorded. Anthropometric measurements and bioimpedance were also measured.

#### Gluteal Fat Biopsy and Proteomic Analysis

A subcutaneous gluteal fat biopsy was obtained from each subject under local anaesthesia, allowing tissue (2 × 1.5 × 1.5 cm) to be harvested. A small section of PVAT was dissected, snap frozen in liquid nitrogen and stored at −80°C for proteomic analysis by Oxford Biomarker Services (Oxford BioTherapeutics Ltd). Protein levels were compared using a label-free mass spectrometric approach. Given our previously published data highlighting the significance of adiponectin in PVAT vasorelaxant function [6], we established a targeted quantification method for adiponectin in these samples using selected reaction monitoring mass spectrometry to determine the relative levels of two adiponectin peptides: GDIGETGVPGAEGPR and IFYNQQNHYDGSTGK (see online suppl. material; see www.karger.com/doi/10.1159/000443885 for all online suppl. material).

#### Biochemical Analysis of Serum

Highly sensitive CRP, leptin, IL-6, TNF-α and adiponectin were assayed by an in-house ELISA technique (hs-CRP, Abcam; remainder, R&D Systems). Insulin levels were measured using a radiolabelling technique as described previously [9]. The homeostasis model assessment (HOMA) [10] was used to estimate beta cell function (HOMA-β) and peripheral insulin sensitivity (HOMA-S).

### Statistical Analysis

The statistical presentation includes paired and unpaired tests. In the case of ordinal tests medians and quartiles are used, and for parametric tests the mean and standard deviation (SD) is presented. Cumulative concentration-response curves were constructed using data obtained by wire myography and analysed using a two-way ANOVA and a Bonferroni post hoc test for each dose. A p value <0.05 was considered statistically significant. Analyses were performed using GraphPad Prism 5 software.

## Results

### Animal Data

Animal Characteristics, BP, NO and PVAT Function

**Fig. 1:**
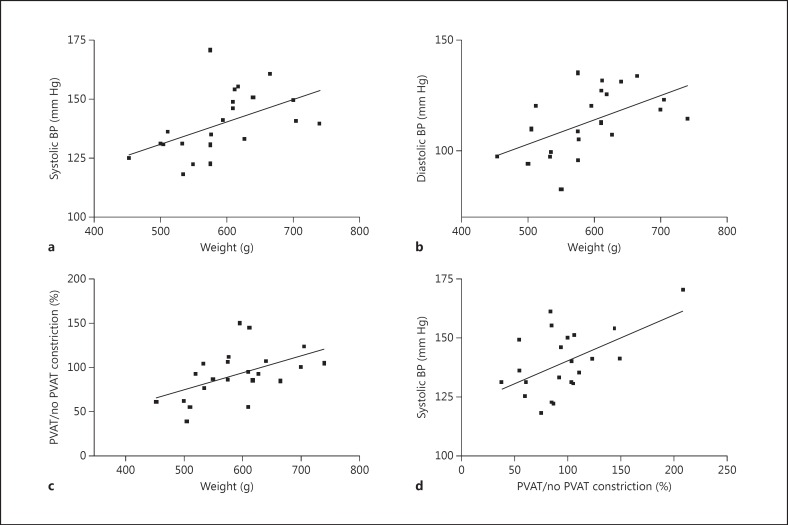
There were significant correlations between weight and both systolic (r = 0.5, p = 0.02; **a**) and diastolic (r = 0.53, p = 0.01; **b**) BP. **c** There was also a significant correlation between weight and damage to PVAT function (r = 0.5059, p = 0.02). **d** The damage to PVAT function also correlated with systolic BP (r = 0.53, p = 0.01).

At the time of sacrifice, the high-fat diet group weighed significantly more than the control group (622 ± 14 vs. 511 ± 13 g; p < 0.001) and developed raised systolic (144 ± 3 vs. 127 ± 3 mm Hg; p < 0.01) and diastolic (118 ± 3 vs. 100 ± 5 mm Hg; p < 0.01) BP. Glucose levels were not significantly different (p = 0.66). PVAT from the control group attenuated vessel contractility to NE (p < 0.0001), but no such effect was observed in the obese group (p = 0.19, data not shown). There were significant correlations between weight and both systolic (r = 0.5, p = 0.02; fig. 1a) and diastolic (r = 0.53, p = 0.01; fig. 1b) BP of the rats at the time of sacrifice.

In order to quantify the ‘PVAT effect’, for a given concentration of NE, constriction of the vessel segment with PVAT was compared to the constriction of vessel segment without PVAT, and this value was presented as a percentage calculation (PVAT effect: constriction of vessel with PVAT/constriction of vessel without PVAT × 100). There was also a significant correlation between PVAT function and weight (r = 0.51, p = 0.02; fig. 1c), as well as with both systolic (r = 0.53, p = 0.01; fig. 1d) and diastolic BP (r = 0.47, p = 0.03; online suppl. fig. 1) at the time of sacrifice.

**Fig. 2:**
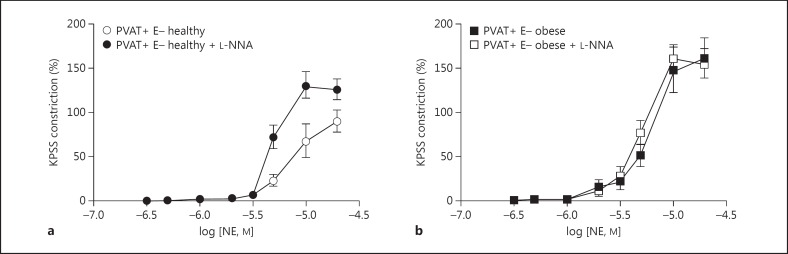
Incubation of PVAT-intact (PVAT+) endothelium-denuded (E-) vessels with the NO synthase inhibitor L-NNA resulted in a significant increase in vessel contractility in healthy vessels (**a**); however, no such phenomenon was observed in obese vessels (**b**).

Incubation of PVAT-intact endothelium-denuded vessels with the NO synthase inhibitor L-NNA resulted in a significant increase in vessel contractility in vessel segments from healthy animals (fig. 2a); however, no such phenomenon was observed in PVAT-intact segments from obese animals (fig. 2b).

#### PVAT Vasorelaxant Function in Obesity Is Damaged Independently of the Endothelium and Incubation with Free Radical Scavengers Restores PVAT Function in Obese Animals

**Fig. 3:**
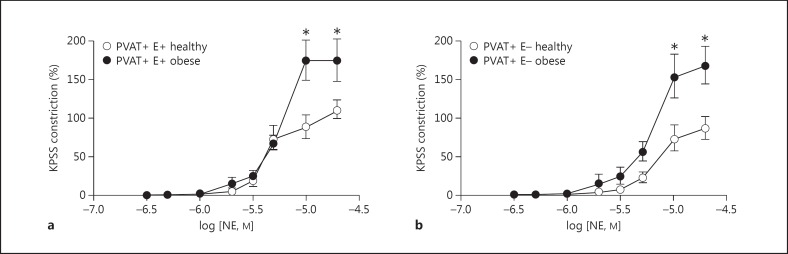
Vessel segments from healthy animals constricted significantly less than segments from obese animals, both when the endothelium was present (E+; p < 0.05, n = 8; **a**) and in endothelium-denuded vessels (E-; p < 0.05, n = 8; **b**). * p < 0.05.

**Fig. 4:**
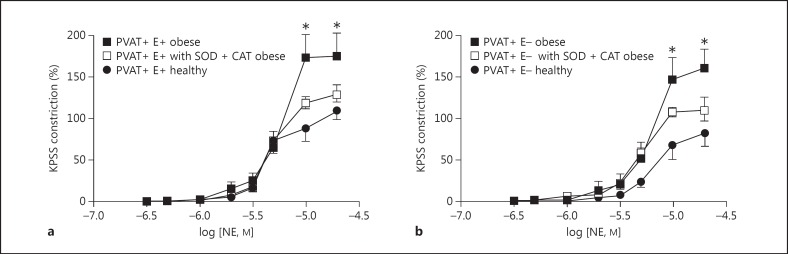
Incubation with SOD and catalase (CAT) resulted in the attenuation of vessel constriction to a similar degree whether the endothelium was intact (E+; **a**) or removed (E-; **b**). * p < 0.05.

Cumulative dose response protocols with NE were performed both with an intact endothelium and after its removal. Vessel segments from healthy animals constricted significantly less than segments from obese animals, both when the endothelium was present (p < 0.05, n = 8; fig. 3a) and in endothelium-denuded vessels (p < 0.05, n = 8; fig. 3b). Incubation with SOD and catalase resulted in the attenuation of vessel constriction to a similar degree, whether the endothelium was intact (fig. 4a) or removed (fig. 4b).

### Human Data

#### Patient Demographics

The obese individuals had a significantly greater waist circumference and significantly higher BMI, systolic BP, HDL (high-density lipoprotein), total cholesterol, HbA_1C_, leptin, insulin and glucose levels (p < 0.05), as well as significantly lower HOMA-S (p < 0.01) and a lower circulating adiponectin level. However, triglycerides, resistin, TNF-α, IL-6 and HOMA-β were not significantly different (table 1).

#### Downregulation of SOD, Peroxiredoxin-1 and Adiponectin in Human PVAT

Proteomic analysis revealed 932 peaks present in over half of the samples analysed and these were used for quantification. Following statistical analysis, 67 peptides were found to be significantly different in these tissues. Removal of peptides emanating from blood-derived proteins left 47 significant peptides (table 2). Levels of the free radical scavengers SOD [Cu-Zn] (SOD1; fold change −2.42, p = 0.02) and peroxiredoxin-1 (PRDX1; fold change −2.15, p = 0.05) were found to be significantly lower in the obese than the lean volunteers. Adiponectin (two peptides: GDIGETGVPGAEGPR and IFYNQQNHYDGSTGK) levels in PVAT were lower in obese patients than in the lean volunteers, with an average fold change of 2.1 (p < 0.05; online suppl. fig. 2).

## Discussion

The present study investigated the effects of obesity on PVAT function and assessed the contribution of NO and endothelium in this process. We have previously shown that in healthy individuals PVAT exerts a vasorelaxant effect on its adjacent small vessel, and that this is not observed in obesity [6]. Moreover, we have shown that this vasorelaxant effect is restored following significant weight loss [11]. Here, we present four novel findings. First, using proteomic analysis, we showed that levels of adiponectin and the free radical scavengers SOD and peroxiredoxin-1 are significantly lower in the PVAT from obese individuals than in that from healthy controls. Second, the obesity-induced damage to PVAT function is independent of the endothelium, and the PVAT vasorelaxant effect is restored using free radical scavengers independent of the endothelium. In healthy animals, there was preservation of the PVAT vasorelaxant capacity after removal of the endothelium, confirming observations made by Gao et al. [12]. Third, NO from PVAT is an important endothelium-independent contributor to the PVAT vasorelaxant function in healthy tissue, but is not observed in obesity. Fourth, PVAT function correlates with systemic BP, and obesity-induced damage to the PVAT vasorelaxant capacity correlates with a rise in BP in our rodent model.

In keeping with increased inflammation in adipose tissue, our proteomic data show that levels of the free radical scavengers SOD1 and peroxiredoxin-1 are significantly lower in obese patients than in healthy lean volunteers. Macrophages present within PVAT contribute to the inflammatory environment by secreting superoxide anions. We know that in obesity there is an increase in macrophage numbers and that following weight loss there is a decline in the number of macrophages within PVAT [8]. More recently, we have shown that the presence and activation of macrophages in adipose tissue are fundamental for the increase in contractility in arteries with perivascular fat following the induction of inflammation [13]. The role of elevated levels of superoxide anions produced by macrophages within the PVAT needs further exploration; however, Gao et al. [14] have shown that PVAT enhances the arterial contractile response to perivascular nerve stimulation via the generation of superoxide anions. SOD converts the highly volatile superoxide anion into hydrogen peroxide, which is then broken down into oxygen and water with the help of catalase, glutathione peroxidase and peroxiredoxin enzymes. Peroxiredoxin-1 resides within the cytoplasm and is the most abundant of the peroxiredoxins [15]. The catalytic efficiency of the peroxiredoxins is less than that of glutathione peroxidase and catalase [16], but a reduction in their levels may lead to a possible reduction in the rate of H_2_O_2_ catabolism and its accumulation in cells with its consequent effects on vascular tone [16]. In the present study, we have shown that in obesity we can rescue the PVAT vasorelaxant effect by incubating the obese PVAT with the free radical scavengers SOD and catalase, and that this is not endothelium dependent.

Gil-Ortega et al. [17] have previously shown that after 8 weeks on a high-fat diet, mouse mesenteric PVAT possesses greater NO bioavailability than controls, and have described this as an adaptive overproduction of the vasorelaxant molecule in early diet-induced obesity. In keeping with their findings, we confirm that, in a healthy state, PVAT vasorelaxant function is partly dependent on NO present within the PVAT itself, independent of the endothelium. However, after 3 months on the high-fat diet, our functional data show a significant reduction in NO bioavailability within PVAT, thus suggesting that the proposed adaptive overproduction of NO is no longer observed once the animals remain on the diet for longer.

Finally, with our rodent model, we have shown for the first time that damage to PVAT vasorelaxant function correlates with a rise in BP. This remains to be explored further using human protocols.

In summary, we have presented further evidence for obesity-induced PVAT damage and its reversibility, as well as correlations between PVAT function, weight and BP in a rodent model of obesity.

## Disclosure Statement

There are no conflicts of interest to declare.

## Figures and Tables

**Table 1:** Demographics of the study participants

	Control participants	Patients with obesity	p
Age, years	51 ± 3.6	52 ± 4	0.95
Waist, mm	920 ± 37	1,140 ± 70	<0.001
BMI	23.9 ± 0.7	35.5 ± 5.2	<0.001
Triglyceride, mmol/l	1.6 ± 0.06	0.52 ± 0.03	0.64
HDL, mmol/l	1.4 ± 0.06	1.2 ± 0.07	0.03
Glucose, mmol/l	5.1 ± 0.1	5.9 ± 0.3	0.01
Systolic BP, mm Hg	135 ± 5	137 ± 3	0.75
Diastolic BP, mm Hg	82 ± 2	80 ± 4	0.67
Cholesterol/HDL ratio	4 ± 0.2	4.2 ± 0.3	0.85
Total cholesterol, mmol/l	5.91 ± 0.31	4.79 ± 0.21	<0.01
HbA_1C_, %	5.7 ± 0.2	6.4 ± 0.1	0.03
Fat, kg	19.7 ± 1.8	38 ± 2.7	<0.001
Impedance	567 ± 22	493 ± 18	0.02
Leptin, μg/l	9.67 ± 2.5	40.4 ± 4.9	<0.001
Resistin, μg/l	9.2 ± 1.2	7.8 ± 0.9	0.35
TNF-α, pg/ml	4.14 ± 0.7	5.21 ± 1.6	0.58
IL-6, pg/ml	2.1 ± 0.3	2.6 ± 0.8	0.63
Adiponectin, mg/l	2.57 ± 0.3	1.72 ± 0.8	0.04
Insulin, mU/l	7.2 ± 1.7	18.3 ± 2.7	<0.01
HOMA-β, %	90.15 ± 10.9	118.8 ± 15.5	0.2
HOMA-S, %	132.4 ± 24.7	49.9 ± 8.3	<0.01

**Table 2:** Proteomic analysis of PVAT

Protein name	Fold change	p ≤ 0.05
DERPC	−9.59	0.05
Parathymosin	−7.18	0.04
Fibrinogen beta chain precursor	−5.01	0.05
Histone H2B.c (H2B/c) * peptide sequence 1	−4.95	0.02
Fibrinogen gamma chain precursor	−4.63	0.03
Serum albumin precursor	−4.47	0.02
Hemoglobin subunit delta * peptide sequence 1	−4.29	0.03
Annexin A2 isoform 1 * peptide sequence 1	−4.02	0.02
Alpha 2 globin variant * peptide sequence 1	−4.02	0.02
Sorting nexin-13	−3.83	0.05
Annexin A2 isoform 1 * peptide sequence 2	−3.74	0.02
Haptoglobin precursor	−3.12	0.01
Histone H2B.c (H2B/c) * peptide sequence 2	−2.93	0.01
Hemoglobin subunit delta * peptide sequence 2	−2.89	0.05
Protein S100-A6	−2.84	0.02
Lumican precursor	−2.7	0.02

Serum albumin precursor * peptide sequence 1	−2.67	0.02
Serum albumin precursor * peptide sequence 2	−2.47	0.04
Hemoglobin beta chain * peptide sequence 1	−2.47	0.04
Superoxide dismutase	−2.42	0.02
Annexin A2 isoform 1 * peptide sequence 3	−2.42	0.02
Alpha 2 globin variant * peptide sequence 2	−2.42	0.02
Vimentin * peptide sequence 1	−2.37	0.004
Hemoglobin beta chain * peptide sequence 2	−2.24	0.03
Collagen alpha 2(I) chain precursor * peptide sequence 1	−2.2	0.01
Profilin-1 (profilin I)	−2.16	0.03
Peroxiredoxin-1	−2.15	0.05
Collagen alpha 2(I) chain precursor * peptide sequence 2	−2.14	0.03
Ubiquitin	−2.10	0.01
Adiponectin	−2.1	0.02

A negative fold change indicated a lower abundance of the protein in the obese than in the control group.
